# Lipocalin-2, glucose metabolism and chronic low-grade systemic inflammation in Chinese people

**DOI:** 10.1186/1475-2840-11-11

**Published:** 2012-01-31

**Authors:** Ying Huang, Zhen Yang, Zi Ye, Qin Li, Jie Wen, Xiaoming Tao, Lili Chen, Min He, Xuanchun Wang, Bin Lu, Zhaoyun Zhang, Weiwei Zhang, Shen Qu, Renming Hu

**Affiliations:** 1 Institute of Endocrinology and Diabetology, Huashan hospital, Shanghai Medical College, Fudan University, Shanghai, China; 2 Department of Endocrinology, Shanghai tenth people's hospital, School of medicine, Tongji University, Shanghai, China; 3 Department of Endocrinology, Xinhua hospital, School of medicine, Shanghai Jiaotong University, Shanghai, China

**Keywords:** Lipocalin-2, Impaired glucose regulation, Type 2 diabetes, Insulin resistance

## Abstract

**Background:**

Lipocalin-2 is a novel adipokine with connection to insulin resistance. In this study, we aimed to investigate the association of serum lipocalin-2 with glucose metabolism and other metabolic phenotype in a large-scale Chinese population.

**Methods:**

We evaluated serum lipocalin-2 in a cross-sectional sample of 2519 Chinese aged from 50 to 82 year in a Shanghai downtown district by ELISA. Glucose, insulin, lipid profile, inflammatory markers, and adipokines were also measured.

**Results:**

Serum lipocalin-2 was significantly higher in subjects with isolated impaired fasting glucose, isolated impaired glucose tolerance, combined impaired fasting glucose/impaired glucose tolerance and newly-diagnosed type 2 diabetes than in those with normal glucose regulation. Lipocalin-2 elevation was clearly associated with a higher risk for impaired glucose regulation (OR 1.30 for each 10 ng/ml increase in serum lipocalin-2, 95% CI 1.23-1.62, *p *= 0.009) after adjustment of age, gender, smoking, alcohol drinking, family history of diabetes, serum CRP, serum adiponectin, serum CXCL5, HOMA-IR, BMI, and waist/hip ratio. The OR for participants with impaired glucose regulation and type 2 diabetes was 1.31 (95% CI 1.21-1.69, *p *< 0.001).

**Conclusions:**

Our findings suggest that elevated serum lipocalin-2 is closely and independently associated with impaired glucose regulation and type 2 diabetes.

## Background

Obesity is a major risk factor for insulin resistance. It is well known that adipocytes could secrete a variety of biologically adipokines, which are thought to contribute to the development of insulin resistance and cardiovascular disease [1-3]. Lipocalin-2, a recently identified adipokine with high levels of expression and secretion in the white adipose tissue, seems to affect glucose metabolism and insulin sensitivity.

Lipocalin-2, also known as neutrophil gelatinase-associated lipocalin, neu-related lipocalin, siderocalin, uterocalin, and 24p3, belongs to the lipocalin super family. It shows high affinity to several small hydrophobic ligands such as retinoids, fatty acids, pheromones, steroids and iron [4-6]. Lipocalin-2 was found expressed in kidney, brain, lung, liver, neutrophils, adipocytes as well as macrophages and was involved in varieties of biological functions such as apoptosis, tumorigenesis and innate immunity [7-9].

Recent studies in animal models showed that lipocalin-2 also plays an important role in systemic insulin sensitivity and glucose homeostasis [10,11]. Lipocalin-2 suppression could attenuate aging and obesity-induced insulin resistance [12]. In human studies, it was reported that lipocalin-2 levels were elevated in both circulation and adipose tissue of obese people. Moreover, circulating lipocalin-2 concentration was positively correlated with insulin resistance index and inflammatory markers [11,13]. Furthermore, elevated serum lipocalin-2 levels were also observed among diabetic patients, and this increase could be reversed by the insulin-sensitizing drug rosiglitazone [11]. However, evidence from large-scale populations about the relationship between lipocalin-2 and glucose metabolism, insulin resistance and chronic low-grade systemic inflammation is scarce.

To better evaluate the role of lipocalin-2 in glucose metabolism, insulin resistance and chronic Low-grade systemic inflammation, we explored the association of serum lipocalin-2 levels with chronic inflammatory marker, insulin resistance and glucose metabolism states in a large-scale Chinese population. We examined lipocalin-2 distribution in 2519 middle-aged and older Chinese subjects in a cross-sectional study and explored the association of serum lipocalin-2 levels with glucose metabolism as well as other metabolic parameters.

## Methods

### Study population

The design and recruitment of the population-based cross-sectional study has been described in detail elsewhere [14]. In 2007 China launched a national incidence trends of metabolic syndrome study. The data presented in this article are partially based on subsamples from Shanghai in eastern China (total 2598 subjects). All studied individuals came from the Simenerlu community of the Jingan District and the Jiangninglu community of the Putuo District in Shanghai, China. A multistage stratified cluster sampling method was used to select subjects from these two communities. Only one participant (> 50 year) was randomly selected from each household. In these 2598 subjects, 79 participants have previously diagnosed type 2 diabetes, all the others received the oral glucose tolerance test and 392 are newly diagnosed type 2 diabetes. In our study, all studied individuals were of southern Han Chinese ancestry and were residing in the metropolitan area of Shanghai aged 50-82 years. Briefly, a total of 2598 eligible participants (1106 men and 1492 women) were recruited. After excluding those who had previously diagnosed as type 2 diabetes and treated (n = 79), 2,519 individuals were eligible for the present analysis. Written consent was obtained from all the participants. The study was approved by the institutional review broad of Huashan Hospital.

### Data collection

A standardized questionnaire was used by trained physicians to collect information such as age, sex, smoking (yes/no), alcohol drinking (yes/no), and self-reported diabetes, hypertension, dyslipidemia. According to participants' responses to the corresponding questions, family history of diabetes was classified as yes or no.

All subjects were assessed after overnight fasting for at least 10 h. The details of anthropometric measurements including height, weight, waist circumference, hip circumference and blood pressure were carried by trained physicians. BMI was calculated as weight in kilograms divided by the square of height in meters and categorized as normal weight (< 24.0 kg/m^2^), or overweight (24-28 kg/m^2^), or obesity (≥ 28.0 kg/m^2^), according to the criteria for Chinese individuals [15].

### Laboratory measurements

Peripheral venous blood samples were collected. The fasting glucose, glucose 2 h after oral glucose tolerance test, total cholesterol, triglycerides, LDL-cholesterol and HDL-cholesterol were measured on an automatic analyzer (Hitachi 7080). Fasting insulin was determined by radioimmunoassay (Linco Research, St. Charles, MO). Insulin resistance was estimated using homeostasis model assessment index-insulin resistance (HOMA-IR).

Type 2 diabetes and prediabetes were defined by 2005 ADA criteria [16]. A fasting glucose level lower than 5.6 mmol/l and a 2 h OGTT plasma glucose level below 7.8 mmol/l were defined as normal glucose regulation (NGR). Impaired fasting glucose (IFG) and impaired glucose tolerance (IGT) were defined as fasting glucose 5.6 to 6.9 mmol/l and 2 h OGTT plasma glucose 7.8 to 11.0 mmol/l respectively. Of 2519 participants, 392 had type 2 diabetes (screen-detected and treatment-naive), 335 had isolated-IFG (all screen-detected and treatment-naive), 353 had isolated-IGT (all screen-detected and treatment-naive), 296 had IFG + IGT (all screen-detected and treatment-naive) and 1143 had NGR.

### Measurements of serum CRP, adiponectin, CXCL5 and lipocalin-2

The serum CRP, adiponectin, CXCL5 and lipocalin-2 were determined in duplicate by ELISA with Duoset kit (DY1707, DY1065, DY254 and DY1757, R&D Systems) as recommended by the manufacturer. The ELISA system had an intraassay coefficient of variation of 3.1-9.5% and an interassay coefficient of variation of 4.3-10.7%, respectively.

### Statistical analysis

Normally distributed data were expressed as means ± SD, whereas variables with a skewed distribution were reported as median (interquartile range) and log transformed to approximate normality before analysis. Categorical variables were represented by frequency and percentage. Univariate and multivariable stepwise regression analysis were used to investigate the association of serum lipocalin-2 with clinical and biochemical characteristics. The stepwise regression procedure was used to obtain determinants of serum lipocalin-2, potential confounding variables including age, gender, smoking, alcohol drinking, family history of diabetes, serum CRP, serum adiponectin, serum CXCL5, HOMA-IR, BMI, waist circumference, and waist/hip ratio were controlled in the regression models. To study the association of impaired glucose regulation (IGR) with lipocalin-2, we defined subjects with normal glucose regulation as 0 (n = 1143), and impaired glucose regulation as 1 (n = 984), and excluded type 2 diabetic patients from logistic regression analysis. For the association of combined impaired glucose regulation and type 2 diabetes with lipocalin-2, we defined subjects with normal glucose regulation as 0 (n = 1143) and combined impaired glucose regulation and type 2 diabetes as 1 (n = 1376) in the logistic regression analyses. Homogeneity of groups was determined when the means showed significant differences. Means of these groups were compared by the Student-Newman-Keuls method. All statistical analysis was performed with the SPSS Statistical Package (version 13.0; SPSS Inc., Chicago, IL). A p value of less than 0.05 was considered to be statistically significant.

## Results

### General characteristics of the study participants

Our study involved 1143 subjects with NGR and 392 with newly diagnosed type 2 diabetes (Table 1). Among those with impaired glucose regulation, 335 had isolated IFG, 353 had isolated IGT, and 296 had IFG + IGT. There was no significant difference in sex distribution, alcohol drinking and serum LDL-c levels between these groups. The NGR and diabetes groups had the most favorable and unfavorable metabolic profiles, respectively. There was no significant difference in waist circumference, waist/hip ratio, and serum triacylglycerol levels between groups with isolated IFG, isolated IGT and IFG + IGT.

**Table 1:** Characteristics of the study population

Characteristics	NGR (1)	IGR				*P *value	Homogeneity of groups
		**Isolated IFG (2)**	**Isolated IGT (3)**	**IFG+IGT (4)**	**Type 2 diabetes (5)**		

n	1143	335	353	296	392	-	-
Male, n (%)	502 (43.9)	121 (36.1)	164 (46.5)	116 (39.2)	173 (44.1)	0.55	-
Age (years)	57.3 ± 75.69	58.2 ± 7.0	60.8 ± 8.4	60.2 ± 8.5	60.9 ± 8.3	< 0.001	(1,2) (3,4) (3,5)
Current smoking, n (%)	211 (18.5)	92 (27.5)	79 (22.4)	58 (19.6)	117 (29.8)	0.25	-
Alcohol drinking, n (%)	135 (11.8)	41 (12.2)	46 (13.0)	29 (9.8)	57 (14.5)	0.89	-
BMI (kg/m2)	24.2 ± 3.3	24.7 ± 3.4	25.1 ± 3.2	25.4 ± 3.5	25.8 ± 3.5	< 0.001	(1) (2) (3,4) (4,5)
Waist circumference (cm)	81.3 ± 8.5	83.3 ± 9.7	85.1 ± 8.5	85.9 ± 9.9	87.3 ± 9.0	< 0.001	(1) (2,3,4) (4,5)
Waist/hip ratio	0.85 ± 0.06	0.87 ± 0.07	0.88 ± 0.07	0.89 ± 0.10	0.89 ± 0.06	< 0.001	(1) (2,3,4) (4,5)
Fasting plasma glucose (mmol/l)	5.3 ± 0.5	6.2 ± 0.3	5.5 ± 0.4	6.0 ± 0.4	7.4 ± 2.4	< 0.001	(1) (2,4) (3) (5)
2 h OGTT plasma glucose (mmol/l)	5.9 ± 0.9	6.1 ± 0.9	9.1 ± 0.9	9.2 ± 1.0	14.6 ± 4.1	< 0.001	(1,2) (3,4) (5)
Fasting serum insulin (μU/ml)	6.86 (4.83-9.94)	6.58 (3.97-9.69)	6.65 (4.47-9.77)	6.54 (4.38-9.62)	7.68 (4.92-11.68)	< 0.001	(1) (2,3,4) (4,5)
HOMA-IR	1.56 (1.06-2.39)	1.41 (0.65-2.22)	1.54 (0.95-2.31)	1.39 (0.74-2.33)	1.93 (1.00-3.32)	< 0.001	(1) (2,3) (4,5)
Systolic blood pressure (mmHg)	126 ± 17	130 ± 17	132 ± 17	135 ± 18	137 ± 18	< 0.001	(1) (2,3,4) (4,5)
Diastolic blood pressure (mmHg)	79 ± 9	81 ± 10	82 ± 11	83 ± 10	83 ± 10	< 0.001	(1) (2,3, 4,5)
Triglycerides (mmol/l)	1.26 (0.90-1.81)	1.40 (0.92-1.95)	1.45 (0.96-2.13)	1.35 (0.95-2.12)	1.57 (1.10-2.28)	< 0.001	(1) (2,3,4) (5)
Total cholerterol (mmol/l)	5.38 ± 1.04	5.32 ± 0.93	5.54 ± 1.07	5.34 ± 1.07	5.59 ± 1.06	0.03	(1,2,3, 4) (5)
LDL cholesterol (mmol/l)	3.13 ± 0.78	3.11 ± 0.69	3.22 ± 0.90	3.18 ± 0.91	3.29 ± 0.87	0.21	(1,2,3, 4) (5)
HDL cholesterol (mmol/l)	1.45 ± 0.51	1.41 ± 0.32	1.35 ± 0.33	1.28 ± 0.31	1.30 ± 0.62	< 0.001	(1,2) (3,4,5)
CRP (μg/ml)	2.41 (1.11-4.95)	2.35 (1.18-4.99)	3.58 (1.61-6.56)	2.73 (1.37-5.48)	3.53 (1.62-7.22)	< 0.001	(1) (2) (3,4,5)
CXCL5 (pg/ml)	1.7 ± 1.3	1.8 ± 1.3	1.9 ± 1.4	1.8 ± 1.2	1.9 ± 1.9	0.03	(1) (2,3, 4,5)
Adiponectin (μg/ml)	10.42 (7.19-14.62)	9.93 (6.58-13.88)	9.47 (6.71-13.62)	10.11 (7.08-13.84)	9.04 (6.12-12.55)	< 0.001	(1) (2,3, 4,5)
Lipocalin-2 (ng/ml)	69.2 ± 7.8	83.5 ± 8.9	92.4 ± 10.7	83.1 ± 8.6	89.2 ± 8.3	< 0.001	(1) (2,3, 4,5)

Data are means ± SD, n (%), or median (interquartile range)

### Determinants of serum lipocalin-2 concentrations

Body mass index, waist circumference, log_10 _fasting serum insulin, log_10 _HOMA-IR, total cholesterol, HDL-cholesterol and serum CXCL5 levels were independent determinants for serum lipocalin-2 concentrations in the stepwise linear regression analysis (Table 2). In univariate analysis, serum lipocalin-2 levels were closely associated with serum C-reactive protein (r = 0.131, *p *< 0.001). However, serum C-reactive protein was not an independent determinant for serum lipocalin-2 (*p *> 0.05).

**Table 2:** Univariate and stepwise regression analysis with serum lipocalin-2 concentration as a dependent variable

Co-variable	Univariate			Stepwise	
	
	r	β(SEM)	*p *value	β(SEM)	*p *value
Age (years)	0.000	-0.002 (0.001)	0.497	-	-
Sex (male = 1, female = 2)	0.086	0.033 (0.020)	0.001	-	-
Body mass index (kg/m2)	0.058	0.015 (0.004)	0.018	0.160 (0.004)	< 0.001
Waist circumference (cm)	0.118	0.000 (0.007)	< 0.001	0.105 (0.001)	0.012
Waist/hip ratio	0.081	0.151 (0.580)	0.005	-	-
Fasting plasma glucose (mmol/l)	0.107	0.011 (0.009)	< 0.001	-	-
2h OGTT plasma glucose (mmol/l)	0.066	0.005 (0.003)	0.009	-	-
Log10 fasting serum insulin (μU/ml)	0.263	0.140 (0.050)	< 0.001	0.105 (0.045)	0.007
Log10 HOMA-IR	0.291	0.123 (0.031)	< 0.001	0.209 (0.025)	< 0.001
Log10 triacylglycerol (mmol/l)	0.134	0.054 (0.053)	< 0.001	-	-
Total cholesterol (mmol/l)	-0.087	0.031 (0.019)	< 0.001	-0.075 (0.008)	0.006
HDL- cholesterol (mmol/l)	-0.181	-0.041 (0.037)	< 0.001	-0.092 (0.026)	0.002
LDL- cholesterol (mmol/l)	-0.059	0.009 (0.020)	0.016	-	-
Systolic blood pressure (mmHg)	0.081	0.001 (0.001)	0.002	-	-
Diastolic blood pressure (mmHg)	0.101	0.000 (0.001)	< 0.001	-	-
CRP (μg/ml)	0.131	0.021 (0.021)	< 0.001	-	-
CXCL5 (pg/ml)	0.076	0.084 (0.026)	0.003	0.08 (0.025)	0.002
Adiponectin (μg/ml)	-0.095	0.015 (0.035)	< 0.001	-	-

β, Regression coefficient; r, Pearson correlation coefficient; *SEM *standard error of mean

### Lipocalin-2 distribution in different glucose metabolism status

**Figure 1:**
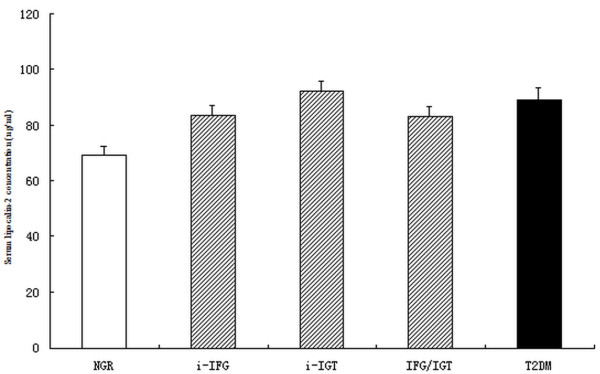
**Adjusted means (± SEM) of serum lipocalin-2 concentrations in subjects with NGR(normal glucose regulation), IGR (isolated IFG[i-IFG], isolated IGT[i-IFGT] and IFG + IGT) and type 2 diabetes (T2DM)**. The covariables included age, gender, BMI and family history of diabetes. Subjects with type 2 diabetes and IGR (i-IFG, i-IGT and IFG + IGT) had higher concentrations of lipocalin-2 than those with NGR (both *p *< 0.01). There was no statiscal difference among the subgroups of IGR (*p *= 0.29) and between the IGR and type 2 diabetes (*p *= 0.37).

Serum lipocalin-2 levels were significantly associated with fasting plasma glucose (r = 0.107, *p *< 0.001) and 2 h OGTT plasma glucose (r = 0.066, *p *= 0.009) in total population. Serum lipocalin-2 concentrations were significantly higher in subjects with isolated IFG, isolated IGT, IFG + IGT, and newly diagnosed type 2 diabetes compared with those of subjects with NGR (83.5, 92.4, 83.1, 89.2 ng/ml vs 69.2 ng/ml, respectively, all *p *< 0.01) after adjustment for age, gender, BMI, and family history of diabetes. There was no statistical difference between the four groups with glucose metabolism dysregulation (all *p *> 0.05, Figure 1).

Remarkably, as presented in Table 3, elevated lipocalin-2 was associated with increased risk of hyperglycemia. Elevated serum lipocalin indicated high risk for impaired glucose regulation (OR 1.30, 95% CI 1.23-1.62, *p *= 0.009) after adjustment for age, gender, smoking, alcohol drinking, family history of diabetes, serum CRP, serum adiponectin, serum CXCL5, HOMA-IR, BMI, waist/hip ratio, serum triacylglycerol, total cholesterol, HDL-cholesterol and LDL-cholesterol. The OR for subjects with impaired glucose regulation and type 2 diabetes was 1.31 (95% CI 1.21-1.69, *p *< 0.001).

**Table 3:** The risk of impaired glucose regulation and type 2 diabetes associated with a 10 ng/ml increase in serum lipocalin-2

Model	IGR (n = 984)	***p *value**^**a**^	impaired glucose regulation and type 2 diabetes (n = 1376)	***p *value**^**b**^
Model 1	1.48 (1.34-1.87)	< 0.001	1.55 (1.43-1.97)	< 0.001
Model 2	1.43 (1.31-1.75)	< 0.001	1.47 (1.38-1.85)	< 0.001
Model 3	1.40 (1.31-1.81)	< 0.001	1.41 (1.29-1.81)	< 0.001
Model 4	1.35 1.24-1.65)	< 0.001	1.38 (1.25-1.81)	< 0.001
Model 5	1.30 (1.23-1.62)	0.009	1.31 (1.21-1.69)	< 0.001

Values are ORs (95% CI)

Model 1: Unadjusted. Model 2: Adjusted for age, sex, smoking (yes/no), alcohol drinking (yes/no), family history of diabetes (yes/no). Model 3: Further adjusted for HOMA-IR, BMI, waist/hip ratio and adiponectin. Model 4: Further adjusted for serum triacylglycerol, total cholesterol, HDL- and LDL-cholesterol. Model 5: Further adjusted for inflammatory factors (CRP and CXCL5). ^a^For the risk of IGR, we defined subjects with normal glucose regulation as 0 (n = 1143) and IGR as 1 (n = 984), excluding type 2 diabetes (n = 392) from the analysis. ^b^For the risk of combined IGR and type 2 diabetes, we defined subjects with normal glucose regulation as 0 (n = 1143) and combined IGR and type 2 diabetes as 1 (n = 1376).

## Discussion

In this study, we found a strong correlation between serum lipocalin-2 levels and impaired glucose regulation as well as type 2 diabetes in a middle-aged and elderly Chinese population. Moreover, this association is independent of potential confounders.

Lipocalin-2 has been reported to be elevated in certain metabolic disorders, including obesity, polycystic ovary syndrome and type 2 diabetes [11,13,17]. In this study, we also observed serum Lipocalin-2 elevation in both type 2 diabetes and impaired glucose regulation. We speculate that lipocalin-2 might be involved in the regulation of glucose metabolism. This hypothesis is supported by several lines of evidence. First, lipocalin-2 knockout mice show significantly decreased fasting glucose and insulin levels and improved insulin sensitivity compared with their wild-type littermates [12]. Second, Yan et al. demonstrated that lipocalin-2 could partially block the suppressive effects of insulin on glucose production and glucose 6-phosphatase expression in hepatocytes. Finally, cells with reduction of lipocalin-2 showed elevated glucose uptake [10].

Pancreatic β-cell dysfunction and insulin resistance are hallmarks of type 2 diabetes. We found that fasting serum insulin and HOMA-IR were clearly associated with lipocalin-2 even in stepwise regression analysis, suggesting that this protein might be an indicator for β cell function and insulin resistance in humans. Lipocalin-2 has been proposed to be an iron delivery protein [18]. Lipocalin-2 delivers iron to the cytoplasm. Iron-loaded lipocalin-2 increases intracellular iron. Iron excess might result in β cell oxidative stress and impaired insulin secretary capacity [19]. Iron deficiency has been related to improved insulin sensitivity in animal models [20,21]. Epidemiologic studies also have confirmed the association between iron overload and peripheral insulin resistance [22]. Furthermore, lipocalin-2 delivers iron to the cytoplasm where it activates ferritin, an iron-responsive gene [18]. Recent study has demonstrated that elevated circulating ferritin concentrations are associated with high risk for type 2 diabetes and metabolic syndrome [23]. Thus, we postulate that Lipocalin-2 might influence β cell function and insulin sensitivity partially through mediation of iron.

Increasing evidences suggest a critical role for PPARγ in the regulation of insulin sensitivity. However, the exact role of lipocalin-2 in obesity-induced insulin resistance remains controversial. Jin et al. observed developed insulin resistance and decreased adipose PPARγexpression in the absence of lipoclain-2 mice, suggesting that lipocalin-2 is a critical selective modulator of PPARγactivation [24]. Jun et al. observed no difference in insulin sensitivity in lipocalin-2 knock out mice on high-fat-diet [25]. However, our study demonstrated that serum concentrations of lipocalin-2 in humans were associated with insulin sensitivity as measured by the HOMA-IR. Consistent with our study, other study have also demonstrated that treatment with the peroxisome proliferator-activated receptor agonist rosiglitazone markedly decreased lipocalin-2 expression in mice and circulating concentrations in both mice and human [11]. Thus, further prospective large population-based studies are needed to investigate the role of lipocalin-2 in obesity--induced insulin resistance.

Visceral fat accumulation has been well-associated with adipose tissue inflammation and insulin resistance [26]. In present study, we found that waist circumference, rather than waist/hip, is one of the independent determinants of serum lipocalin-2 levels. It has been well-documented that waist circumference reflects the amount of visceral adipose tissue, which is closely correlated with the development of metabolic disorders. Hence, the accumulated visceral fat might account for the serum lipocalin-2 elevation during insulin resistance. In line with our hypothesis, some studies have demonstrated that lipocalin-2 is selectively expressed in mouse epididymal fat depot and its expression is highly induced during adipocyte differentiation [27,28]. Moreover, the expression of lipocalin-2 in visceral adipose tissue was found to be correlated with its circulating levels in animal models [13]. Certainly, more work is urgently needed to clarify whether visceral adipose tissue plays a causal role in the elevation of lipocalin-2.

Type 2 diabetes was associated with wide pathophysiological pathways, including inflammation [29]. CRP is a pattern-recognition molecule of innate immunity as an acute-phase reactant and a hallmark of low-grade systemic inflammation. We confirmed the strong and positive association between lipocalin-2 and CRP levels observed in previous studies [11], suggesting elevated serum lipocalin-2 in prediabetes and type 2 diabetes might associate with chronic inflammation. Previous studies have demonstrated that circulating CRP is an independent risk factor for type 2 diabetes and atherosclerosis [30,31]. Yan et al. found that IL-1β and TNFα are both strong inducers of lipocalin-2 production [10]. Another study shows that lipocalin-2 directly stimulates the expression and activity of 12-lipoxygenase as well as TNF-α production in fat tissue. Conversely, these changes were attenuated in lipocalin-2 deficient mice [12]. These findings suggested that lipocalin-2 might play a causal role in the pathogenesis of inflammation. However, lipocalin-2 has recently been claimed to exert antiinflammatory function, and these effects were associated with its modulation of PPARγ activity via direct or indirect mechanisms by inhibiting NFkB activity [27]. In our study we found that CRP is not an independent risk of lipocalin-2. With the cross-sectional nature, we are limited in the ability to evaluate whether elevated lipocalin-2 is a protective mechanism against overactivaion of inflammation or an inflammation inducer.

One of the interesting findings of the present study is that the concentration of lipocalin-2 is also associated with CXC ligand 5 (CXCL5). Elevated CXCL5 levels were strongly and independently associated with insulin resistance [32]. CXCL5 is an adipose tissue derived factor that links obesity with insulin resistance. CXCL5 blocks insulin signaling by activating the Jak2/STAT5/SOCS2 pathway. CXCL5 levels were also demonstrated to have a positive association with inflammatory factor CRP in our previous study [14]. Taken together, lipocalin-2 and CXCL5, as innate immunity indicators, are both serum markers of low-grade systemic inflammatory response and could potentially modulate chronic inflammatory processes.

To our knowledge, this is the first study to investigate the association of serum lipocalin-2 and the development of type 2 diabetes as well as impaired glucose regulation in Chinese. The main strength of our study is that all diabetic participants were newly diagnosed. None had used glucose-lowering treatment or insulin. Hence, the confounding effect of glucose-lowering treatment could be excluded. Anyway, there are still some limitations in this work. First, HbA1c levels were not measured in this study, some patients with diabetes might have been missed. Second, No causal relationship between lipocalin-2 levels and the risk of impaired glucose regulation or diabetes can be drawn, since the study was cross-sectional designed.

## Conclusion

In conclusion, our study indicates that serum lipocalin-2 is independently correlated with impaired glucose regulation and type 2 diabetes. Although the underlying pathophysiological mechanisms remain unclear, it seems that insulin resistance and chronic low-grade systematic inflammation may be involved in this association.

## Competing interests

The authors declare that they have no competing interests.

## Authors' contributions

Conceived and designed the experiments: ZY, RH Performed the experiments: YH, ZY, QL Analyzed the data: ZY Contributed reagents/materials/analysis tools: JW, XT, LC, MH, XW, BL, ZZ, WZ, SQ. Wrote the paper: YH All authors read and approved the final manuscript.
